# The Impact of ^18^F-FDG PET CT Prior to Chemoradiotherapy for Stage III/IV Head and Neck Squamous Cell Carcinoma

**DOI:** 10.5402/2012/636379

**Published:** 2012-03-24

**Authors:** Robin J. D. Prestwich, Priya Bhatnagar, Fahmid U. Chowdhury, Chirag N. Patel, Karen E. Dyker, Catherine Coyle, Mehmet Şen, Andrew F. Scarsbrook

**Affiliations:** ^1^Departments of Nuclear Medicine, St. James's Institute of Oncology, Leeds LS9 7TF, UK; ^2^Departments of Clinical Oncology, St. James's Institute of Oncology, Leeds LS9 7TF, UK; ^3^Departments of Clinical Radiology, St. James's Institute of Oncology, West Yorkshire, Leeds LS9 7TF, UK

## Abstract

*Introduction*. To determine the value of a FDG-PET-CT scan in patients with locally advanced head and neck squamous cell carcinoma (HNSCC) prior to chemoradiotherapy. *Materials and Methods*. Consecutive patients with stage III or IV HNSCC who had undergone a staging FDG-PET-CT scan prior to chemoradiotherapy between August 2008 and April 2011 were included. Clinical details and conventional imaging (CT and/or MRI) were, retrospectively, reviewed, a TNM stage was assigned, and levels of cervical lymph node involvement were documented. This process was repeated with the addition of FDG-PET-CT. Radiotherapy plans were reviewed for patients with an alteration identified on TNM staging and/or nodal level identification with FDG-PET-CT and potential alterations in radiotherapy planning were documented. *Results*. 55 patients were included in the analysis. FDG-PET-CT altered the TNM stage in 17/55 (31%) of patients, upstaging disease in 11 (20%) and downstaging in 6 (11%); distant metastases were identified by FDG-PET-CT in 1 (2%) patient. FDG-PET-CT altered the lymph node levels identified in 22 patients (40%), upclassifying disease in 16 (29%) and downclassifying in 6 (11%). Radiotherapy plans were judged retrospectively to have been altered by FDG-PET-CT in 10 patients (18%). *Conclusions*. The use of FDG-PET-CT potentially impacts upon both treatment decisions and radiotherapy planning.

## 1. Introduction

Locoregional staging for head and neck squamous cell carcinoma (HNSCC) is performed using a combination of clinical examination, examination under anaesthetic and crosssectional imaging with computed tomography (CT) and/or magnetic resonance imaging (MRI). Anatomical imaging is fundamentally limited for detection of malignancy in nonnecrotic lymph nodes which fall below standard size criteria for example, short axis diameter of 10 mm [1]. This is illustrated by the finding that only micrometastases of less than 3 mm were present in 25% of pathologically proven tumour-positive neck dissections [1]. An ultrasound study has suggested that a cutoff of 6 mm may be the optimal compromise between sensitivity and specificity for cervical lymph nodes [2]. The risk of lymph node metastases is dependant upon tumour site, extent, and lateralisation [3]. The sensitivity and specificity of CT and MRI have been estimated at 50–80% and 70–90%, respectively [4, 5]. The failure of conventional staging to detect disease in the clinically node negative neck (cN0) is recognised in treatment protocols by the incorporation of prophylactic therapy to the neck with either a neck dissection or radiotherapy when the risk of nodal metastases is expected to exceed 20% [6].

In the modern era, organ preserving nonsurgical radiotherapy-based approaches to the management of locally advanced HNSCC are increasingly employed [7]. Treatment-related late morbidity can be substantial [8]. The intensity-modulated radiotherapy (IMRT) technique utilises multiple radiation beams of nonuniform intensity derived from inverse planning and is now in routine clinical practice for HNSCC [9]. IMRT allows radiotherapy doses to be accurately delivered with steep dose gradients and lower doses to areas deemed to be at lower risk and minimal dose to other organs. The ability of IMRT to deliver doses with a high degree of conformality has led to interest in the use of IMRT to successfully eliminate sites of tumour involvement whilst sparing normal tissues [10, 11]. Appropriate patient selection and accurate target definition are critical to the success of attempts to spare normal tissues, with the potential for the risk of increased nodal recurrence associated with the steep dose gradients in IMRT [12]. Indeed, quality of radiotherapy has been identified as a major factor in determining outcome of nonsurgical treatment for HNSCC [13]. Reliable localisation of sites of tumour involvement is essential to the success of treatment protocols aimed at limiting late tissue toxicity.

The radiation oncologist frequently has to make difficult judgements based on anatomical imaging to include or otherwise equivocal lymph nodes in the high-dose target volume. Functional imaging techniques offer the potential to provide complementary information to anatomical imaging with CT and MRI to aid in these decisions. ^18^Fluoride-fluorodeoxyglucose positron emission tomography (FDG PET) is a widely used functional imaging technique in oncology. Tumour cells exhibit differential glucose uptake (the “Warburg effect”) as a basis of the identification of cancer [14]. Increased glucose uptake by nonmalignant tissue, commonly in the presence of infection or inflammation, leads to false positive results. Integrated PET-CT, which combines the complementary techniques of PET and CT in a single study, offers the potential to improve upon the inherent size limitations in terms of accurate lymph node identification with MRI and CT, not being limited by formal size criteria [15]. Limitations of PET include issues with regard to scanner resolution, partial volume effects, and the need for accurate coregistration with the CT scan. FDG PET-CT imaging in HNSCC has multiple potential applications including staging, radiotherapy planning, treatment adaptation, response assessment, and recurrence detection [9, 15, 16]. The site of recurrence has been shown to correlate with the baseline sites of FDG-avid disease [17]. FDG PET-CT has been demonstrated to be able to identify metastatic head and neck malignancy in cases where MRI and CT fail to demonstrate disease [18, 19]. The use of FDG PET-CT for staging HNSCC (other than in the setting of cervical lymph node metastases of unknown primary origin) remains controversial, with some authorities not recommending its use in routine staging [9], whilst others support the role of FDG PET-CT for staging locoregional and distant disease [20]. Some studies have suggested that the addition of FDG PET-CT to conventional staging methods improves the accuracy of nodal assessment [21–23]. At the heart of any debate regarding the utility of FDG PET-CT in addition to conventional anatomical imaging is the assessment of the potential impact of the investigation upon patient management. This impact may vary depending upon the patient population, tumour stage, and treatment under consideration.

The aim of this study was to assess the utility of FDG PET-CT as an adjunct to conventional staging methods in patients with locally advanced HNSCC due to undergo primary nonsurgical treatment. The value of FDG PET-CT on identification of levels of nodal involvement was determined to assess whether FDG PET-CT can influence process of defining the radiotherapy target volume.

## 2. Methods

### 2.1. Inclusion Criteria

Formal institutional review board approval was waived for this retrospective study. Consecutive patients between August 2008 and April 2011 who underwent a FDG PET-CT scan for head and neck cancer were obtained from an institutional database. Electronic case notes were used to identify patients who fulfilled the eligibility criteria for the study.

Eligible patients fulfilled all of the following criteria.

1. Histologically confirmed squamous cell carcinoma of the oropharynx, oral cavity, hypopharynx, larynx, or paranasal sinuses.
2. Nasopharyngeal cancer was excluded.
3. Reviewed by specialist head and neck multidisciplinary meeting.
4. TNM stage III or IV prior to FDG PET-CT scan.
5. Decision made prior to FDG PET-CT to proceed with radical nonsurgical treatment (radiotherapy alone or chemoradiotherapy).
6. FDG PET-CT performed prior to commencement of treatment for staging and/or as a baseline for future response assessment.

Baseline demographics were obtained from review of electronic case notes (Patient Pathway Manager, Leeds).

### 2.2. Staging

Conventional staging of locally advanced HNSCC was routinely performed by physical examination and neck palpation, fiberoptic endoscopy, examination under anaesthetic with biopsy where indicated, MRI or contrast-enhanced CT of head and neck region depending upon local protocols, and CT of the thorax. Results were routinely reviewed in a specialist head and neck MDT meeting and a TNM classification, based on all available clinical and radiological data, according to the American Joint Committee on Cancer TNM staging was assigned prior to the FDG PET-CT scan [24]. The method of conventional crosssectional imaging used for staging was recorded from radiology records.

### 2.3. CT Protocol

Contrast-enhanced CT was most commonly performed at our institution after referral from the regional oncology team on a 64-slice CT (Siemens Sensation, Siemens Healthcare, Erlangen, Germany) using a contiguous 1 mm reconstruction following a bolus of 100 mL of iodinated contrast or a 16-slice CT (Siemens 16, Siemens Healthcare) using the same acquisition parameters. The remaining contrast-enhanced CT scans were acquired at one of several referring hospitals on multislice CT systems using similar acquisition parameters.

### 2.4. MRI Protocol

MRI examinations of the head and neck were most commonly performed at our institution using 1.5-Tesla systems (Symphony/Avanto; Siemens, Erlangen, Germany). Scan limits were skull base to clavicles. Standard sequences included axial and coronal T1, axial T2 (fat sat) and coronal STIR, and axial and coronal T1 post-Gadolinium. Sagittal T1 post-Gadolinium was performed for tongue base tumours.

### 2.5. FDG PET-CT Protocol

FDG PET-CT examinations prior to June 2010 were performed on a 16-slice Discovery STE PET-CT scanner (GE Healthcare, Amersham, UK) and from June 2010 on a 64-slice Philips Gemini TF64 scanner (Philips Healthcare, Netherlands). PET acquisition from skull vertex to upper thighs was performed 60 minutes after a 400 MBq dose of intravenous ^18^F-FDG. A silence protocol was employed in the uptake period following tracer injection to minimise physiological tracer activity within the head and neck region. The CT component was performed according to a standardised protocol (without the use of iodinated contrast medium) with the following settings: 140 kV; 80 mAs; tube rotation time 0.5 s per rotation; pitch 6; section thickness 3.75 mm (to match the PET section thickness). Patients maintained normal shallow respiration during the CT acquisition. Images were reconstructed using a standard OSEM algorithm with CT for attenuation correction. Both nonattenuation corrected and attenuation corrected datasets were reconstructed.

### 2.6. Review of Imaging Investigations

Imaging for each patient was retrieved from the institutional picture archiving and communications system (PACS)(IMPAX, AGFA Healthcare, Mortsel, Belgium). PET-CT imaging was reviewed on a specialised PET-CT workstation (XD3, Mirada-Medical, Oxford, UK). Imaging was retrospectively reviewed by two experienced radiologists independently. Visual interpretation was qualitative, with regions with an FDG uptake above background mediastinal blood pool classed as positive. Any discrepancies were discussed in order to reach a consensus view. Clinical information and MRI and/or CT scans were reviewed by the same radiologists in conjunction with radiology reports. Standard size criteria [1] were used to determine lymph node involvement on CT and MRI imaging. Levels of lymph node involvement were documented. This study was intended to determine the incremental rather than independent value of FDG PET-CT. AJCC TNM classification was recorded based upon assessment of the FDG PET-CT scan in conjunction with other prior imaging and clinical information. In the same manner, involved lymph node levels were documented.

Based upon this data, any alterations in the TNM stage and/or levels of lymph node involvement between conventional imaging and PET-CT were documented.

### 2.7. Clinical Impact

#### 2.7.1. Impact upon Treatment Modality/Intent

Management plan prior to FDG PET-CT staging was determined from electronic case notes and MDT records. Alterations in treatment modality/intent of treatment following FDG PET-CT were determined by retrospective review of records.

#### 2.7.2. Impact upon Radiotherapy Planning

During the early part of the study period, patients were treated with a conformal technique involving a parallel opposed pair [25]; this technique would not allow nodal levels within the high-dose volume to receive different doses. IMRT was subsequently implemented into routine clinical practice, facilitating the treatment of different lymph node levels with different doses. Institutional protocols were followed with a radical treatment dose of 70 Gy in 35 fractions over 7 weeks, with lower doses to prophylactic dose regions (54–63 Gy in 35 fractions over 7 weeks).

In general, using either technique, gross tumour volume was included within the high-dose volume, and areas deemed to be at lower risk were treated with prophylactic doses. In patients in whom a discrepancy was noted between the TNM staging and/or lymph node level identification, radiotherapy plans were reviewed to determine whether the lymph node was included in high-dose or prophylactic dose regions. A retrospective qualitative judgement was made as to whether FDG PET-CT altered had no impact or was ignored during the radiotherapy planning process. This was assessed by two experienced clinical oncologists independently, and any disagreements were discussed in order to reach a consensus view.

### 2.8. Statistics

Baseline characteristics and radiological findings were summarised using descriptive statistics.

## 3. Results

55 patients were eligible for inclusion in the study. Median age was 55 (range 28–75). 42/55 (76%) were male. Smoking, alcohol history, and performance status were recorded in 54 patients; 23 were current smokers, 10 exsmokers, and 21 had never smoked. 13 were classed as having a heavy alcohol intake (at least 50 units/week), 36 drank some alcohol (<50 units/week), and 5 did not drink alcohol. World Health Association (WHO) performance status was recorded in 54 patients: WHO PS 0,1,2 in 31, 16, and 7 patients, respectively.

Tumour site, subsite, stage prior to FDG PET-CT, histology, and intended management plan prior to FDG PET-CT are shown in Table 1. The TNM distribution is shown in Table 2. 12 patients were conventionally staged with both MRI and CT examinations, 33 were staged with MRI alone, and 10 with a CT alone, in addition to clinical examination.

### 3.1. TNM Staging Changes Resulting From FDG PET-CT Examination

The median time between CT or MRI staging of the head and neck and the FDG PET-CT scan was 17.5 days (range 4–42). The median maximum SUV of the primary tumour was 13.1 (range 5.3–44) and for lymph nodes 8.7 (range 5.3-44). TNM stage at FDG PET-CT was discordant with staging utilising clinical information and CT and/or MRI in 17 (31%) of patients. The changes are summarised in Table 3. In one patient with locally advanced tonsil cancer, CT thorax demonstrated 5 small (sub-5 mm) indeterminate lung nodules. PET-CT demonstrated FDG uptake in the pulmonary nodules and identifying a contralateral retropharyngeal lymph node; TNM classification was therefore altered from T4N2bMx to T4N2cM1. All other changes to TNM classification were within the nodal classification.

### 3.2. Changes in Identification of Lymph Node Involvement Resulting from FDG PET-CT Examination

**Figure 1:**
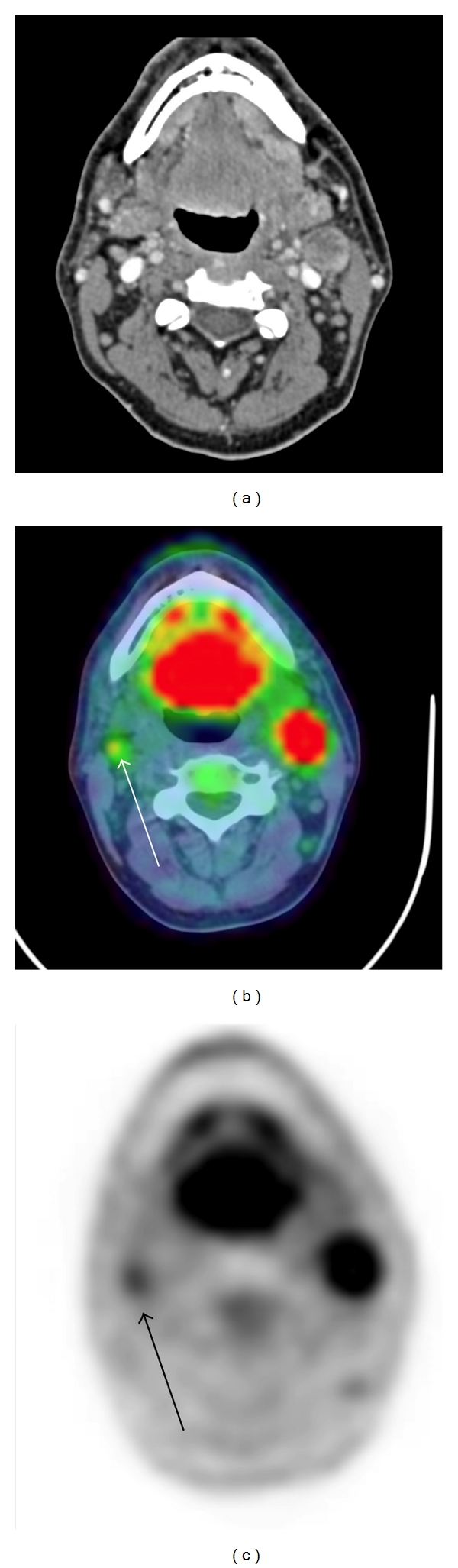
Altered TNM staging and lymph node level identification with FDG PET-CT. T4 squamous cell carcinoma of left tonsil. Diagnostic CT identified T4 N2b disease. FDG PET-CT identified a subcentimetre (SUV_max_ = 3.5) contralateral lymph node, altering staging to T4 N2c. (a) Diagnostic contrast-enhanced CT. (b) Coregistered FDG PET-CT. (c) FDG PET. White arrow = right level II lymph node.

The involvement of neck lymph node levels was determined utilising clinical information and CT and/or MRI imaging. There was discordance in lymph node level involvement on FDG PET-CT and conventional imaging in 22 of 55 (40%) patients (Summarised in Table 4). An example in which FDG uptake was detected in an additional subcentimetre lymph node altering TNM staging and lymph node level identification is shown in Figure 1. Alterations of nodal staging included both upstaging and downstaging. Histological correlation was not performed on these patients.

### 3.3. Upstaging

In 16 patients, FDG PET-CT suggested involvement of an additional lymph node level not identified on other imaging. In 5 of these cases, PET-CT detected FDG-positive contralateral lymph nodes, where no contralateral nodal involvement had been previously demonstrated. In 3 cases, PET-CT showed FDG uptake suggestive of nodal involvement in what had been classed as an N0 neck.

### 3.4. Downstaging

In 6 patients FDG PET-CT downclassified possible lymph node involvement on anatomical imaging on the basis of a lack of FDG avidity. In each of these cases, nonnecrotic lymph nodes identified on the staging CT and/or MRI were regarded as equivocal based upon size criteria. In 5 of these 6 cases, the FDG PET-CT findings altered the staging of an N2c neck to unilateral nodal disease. In the remaining case, an equivocal lymph node was downclassified to an N0 stage.

### 3.5. Impact of FDG PET-CT Findings on Clinical Management

The treatment intention was altered in 1 of 55 patients following FDG PET-CT. This patient, as discussed above, had small indeterminate lung nodules on CT. The demonstration that these were highly likely to be metastatic on FDG PET-CT led to the patient being offered palliative rather than radical radiotherapy. Further clinical and imaging followup is awaited on this patient. There were no alterations in treatment modality as a result of FDG PET-CT. One patient altered his treatment decision and opted for surgery opposed to nonsurgical treatment. Two patients were treated with palliative rather than radical radiotherapy due to a deteriorating performance status and patient preference. In one of these patients FDG PET-CT had altered nodal stage from N2a to N2b although no additional lymph node levels were identified. The 52 remaining patients all received the radical nonsurgical treatment modality that had been proposed prior to the FDG PET-CT.

The radical radiotherapy treatment plans of patients with an alteration in involved neck lymph node levels based on FDG PET-CT (Table 4), excluding patient 18 who was the patient who opted for surgical treatment and patient 22 who had probable lung metastases were retrospectively reviewed to ascertain the impact of FDG PET-CT upon radiotherapy planning (Table 5). 17 of these 20 patients were treated with an IMRT technique, one with unilateral conformal technique and two with a conformal parallel opposed pair.

In the 6 patients with equivocal lymph nodes on conventional imaging which were FDG negative, one patient received unilateral radiotherapy as a result of the FDG PET-CT findings; 2 patients were treated only with prophylactic doses. In these 3 patients, FDG PET-CT was deemed to have altered the radiotherapy plan. One patient received a parallel opposed pair technique, precluding any impact of the FDG PET-CT upon the plan. In the remaining two patients, the FDG PET-CT findings were not utilised as the FDG negative, lymph node regions were treated in the high-dose volume.

**Figure 2:**
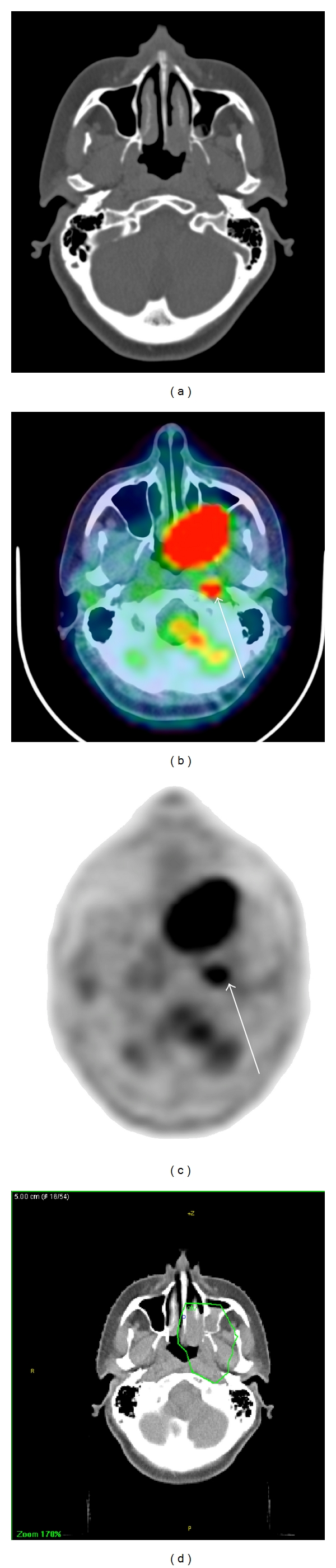
Modification of radiotherapy target volume based on FDG PET-CT. T4 maxillary sinus SCC. Diagnostic CT staged at T4 N0. FDG PET-CT identified an FDG avid (SUV_max_ = 12) left retropharyngeal lymph node altering staging to T4 N1. The radiotherapy clinical target volume incorporates this lymph node. (a) Diagnostic CT. (b) Coregistered FDG PET-CT. (c) FDG PET. (d) Green line = clinical target volume on planning CT scan including FDG avid left retropharyngeal lymph node. White arrow = FDG avid left retropharyngeal lymph node.

In the 14 patients in whom FDG PET-CT identified additional lymph node levels, these nodal levels were included in the high-dose volume in 11 cases. An example of a high-dose clinical target volume including the FDG avid lymph node is shown in Figure 2. A subjective judgement was made that the plan had been altered by FDG PET-CT in 7 of these cases. In 2 cases, the FDG PET-CT findings were not utilised, with FDG avid nodal areas treated to prophylactic doses. In both of these cases identified lymph nodes were subcentimetre in size and showed only mild FDG uptake.

FDG PET-CT demonstrated FDG avid axillary lymph nodes in 2 patients (SUV 6.7 in a 10 mm lymph node in one patient and an SUV of 5.4 in a 17 mm lymph node in the second patient). In both cases ultrasound-guided biopsies demonstrated reactive changes only, and there has been no clinical progression of the axillary lymph node after clinical followup of 14 and 8 months, respectively. Therefore, in these 2 cases, FDG PET-CT generated a false positive result outside of the head and neck region that had required further investigation.

## 4. Discussion

Advances in the ability to accurately identify all sites of disease underpin attempts to improve radiotherapy outcomes for HNSCC. Purely anatomical imaging with CT and MRI is inherently limited in failing to detect nonenlarged malignant lymph nodes. Functional imaging techniques, such as integrated FDG PET-CT, are not limited in the same manner, and their incorporation into existing staging protocols is highly attractive. FDG PET-CT could be used in the radiotherapy planning process to identify the target lymph nodes and/or to be used to contour target tissue. The delineation of target lymph nodes using FDG PET is highly dependent upon the methodology of PET segmentation which remains highly controversial [26, 27].

Evidence with regard to the ability of FDG PET-CT to improve upon the staging of cervical lymph node levels is based upon multiple retrospective and prospective series. Kyzas et al. [28] performed a meta-analysis in which 32 studies with 1236 cases were included with histological verification to attempt to determine the ability of FDG PET-CT to evaluate cervical metastases in HNSCC. Within this meta-analysis, FDG PET had a sensitivity of 79% (95% CI 72–85%) and a specificity of 86% (95% CI 83–89%). Notably, FDG PET only had a sensitivity of 50% (95% CI 37–63%) in the N0 neck. In an attempt to compare these results with conventional imaging, a subanalysis was performed on studies in which both FDG PET and conventional tests were performed. In these studies, FDG PET had a sensitivity and specificity of 80% and 86% versus 75% and 79% for conventional imaging alone. Therefore, FDG PET appears to offer a modest improvement in the neck staging accuracy. Furthermore, in clinical practice the important question is not CT/MRI versus FDG PET-CT, but rather determining what is the incremental benefit of PET-CT in addition to routine imaging including CT/MRI [15]. Kubicek et al. [29] retrospectively reviewed 212 PET scans of patients who went on to receive radiotherapy and report high positive and negative predictive values of 94% and 89%, respectively. The positive and negative predictive value of the test will depend upon the patient population to which it is applied. For example the value of FDG PET-CT in identifying an additional nodal level in an N+ neck to assist radiotherapy planning is likely to be different to the situation in an N0 neck. The ability of FDG PET-CT to stage an N0 neck is currently under investigation in a prospective USA study with pathological correlation, ACRIN 6685 [30].

A series of studies have explored whether the use of FDG PET-CT has any impact upon clinical management of HNSCC. Several series have now demonstrated the impact of FDG PET on management decisions [21, 31–36]. Analysis of a large retrospective series of 123 patients showed a management change in 31% of patients [21]. In a prospective study of 71 patients, FDG PET was reported to alter the TNM stage in 31% of patients (this study did not include pathological validation) [31]. In the largest series, a multicentre prospective study, Lonneux et al. [32] evaluated the addition of FDG PET to conventional staging of HNSCC. Out of 233 patients, FDG-PET and conventional TNM stage were discordant in 100 patients. Histological confirmation was available in 41 of these 100 discordant patients, with additional imaging and clinical followup regarded as “gold-standard” in a further 19 cases. Out of these “confirmed” 60 patients, nodal stage was accurately upgraded in 16 and downgraded in 7. FDG PET was found to be accurate in 67% of patients with confirmed nodal discrepancies. Of the 40 patients lacking “confirmation”, the N stage was upgraded in 28 and downgraded in 11. Therefore, nodal staging discrepancies within this study were common. The study did not report alterations in involvement of nodal levels which did not alter TNM staging. Overall, the study reported that FDG PET altered patient management in 13.7% of patients; this included the detection of distant metastases in 6 patients and an alteration of radiotherapy fields in 8 patients.

It is important to note that within almost all of the studies in the Kyzas et al. [28] meta-analysis and the majority of patients in the other major series [31, 32], FDG PET was single modality rather than integrated PET-CT, which has subsequently been shown to have higher accuracy than FDG PET alone [36–38].

Our series of 55 patients differs from other reported series in that a decision to proceed with primary nonsurgical had been made prior to the FDG PET-CT scan and that patients were restricted to stage III/IV HNSCC. FDG PET-CT was performed both for staging and to provide a baseline for future response assessment. In order to examine the clinical utility of FDG PET-CT, we analysed the data to determine the incremental change to TNM staging and alteration of distribution of lymph node level involvement, in addition to conventional staging. Abnormal areas of FDG avidity were defined as being above background, as previously described by Lonneux et al. [32]. Changes in lymph node level involvement were highlighted to assess the potential consequences to IMRT planning. The time between conventional staging and the FDG PET-CT was short (median 17.5 days). Therefore, disease progression between imaging studies is unlikely to have had a significant impact.

The main limitation of this study is that pathological validation was not performed in any of these patients. This has also been the case in other series [31, 33]. The majority of patients (85%) had oropharynx primary tumours. In our practice these patients are managed without surgical intervention other than for salvage [25, 39]. Although ultrasound-guided fine needle aspiration offers the option of attempting to confirm FDG PET-CT findings, further investigation risks treatment delay and sampling errors especially for small lymph nodes. With increasing experience of FDG PET-CT, a pragmatic approach may be taken whereby histological clarification may not always be required when the imaging findings are categorical and give a high level of confidence for disease involvement. Under these circumstances, invasive sampling may be reserved for cases where the imaging findings are more equivocal.

The retrospective nature of this study is a further potential limitation. This is minimised by the interpretation at separate sittings of both imaging and radiotherapy plans by two radiologists and clinical oncologists, respectively. Although there is the possibility of selection bias of patients undergoing PET-CT, the TNM distribution of these stage III/IV patients is broadly similar to that in our previous report of the outcome of stage III/IV tonsil cancers [25]. PET-CT scans in this series were performed without intravenous contrast. However, the availability of recent contrast-enhanced imaging with CT and/or MRI reduces the likelihood of this significantly reducing the quality of PET-CT scan interpretation.

In these 55 patients, TNM stage was altered by the FDG PET-CT findings in 17 (31%) patients (Table 3). This is consistent with alterations in TNM staging in other series: 40% in Lonneux et al. [32], 34% in Connell et al. [33], and 31% in Scott et al. [31]. The frequency of alteration in the identification of levels of nodal involvement has not been reported in other series. This has potential impact upon IMRT planning in which involved and uninvolved nodal levels will receive different doses. FDG PET-CT in addition to clinical assessment and conventional anatomical imaging altered the identification of nodal levels in 22 of 55 patients (40%) (Table 4). In 16 of these patients, FDG PET-CT demonstrated at least one additional nodal level, whilst in 6 patients potential nodal involvement was downstaged. The major limitation in interpreting these findings is the lack of pathological correlation. FDG PET-CT altered the interpretation of lymph nodes which were small and largely subcentimetre in maximum diameter. In addition, some lymph nodes demonstrated low-grade FDG uptake, and accuracy of staging may have been improved by invasive sampling in such cases. Whilst accepting that histological validation is absent, it is possible to interpret these results in the light of other studies. As discussed, Kyzas et al.[28] showed that ^18^FDG-PET has a higher sensitivity and specificity for lymph node involvement than CT or MRI. In the study by Lonneux et al. [32], FDG PET had a 67% accuracy in nodes where there was a discrepancy with conventional staging. On this basis, the use of FDG PET-CT data in radiotherapy planning for this group of patients with locally advanced disease is likely to improve target definition.

The potential impact on clinical management is difficult to assess in a retrospective series and is inevitably subjective in nature. Only one patient in whom lung metastases were identified had a clear change in treatment intent/modality. In the 6 patients in whom FDG PET-CT downclassified nodal involvement, radiotherapy planning was felt to have been altered in 3 patients (Table 5); in one of these, the lack of FDG uptake in an equivocal lymph node allowed unilateral radiotherapy, whilst in 2 others the downclassified lymph nodes were treated with prophylactic radiotherapy doses. In 11 of the 14 cases in which an additional lymph node level was identified by FDG PET-CT (Table 5), this nodal level was included in the high-dose volume. In 7 of these cases, FDG PET-CT was felt to have altered the radiotherapy plan. Therefore, FDG PET-CT appeared to have affected the radiotherapy planning process with regard to the lymph node target in a total of 10/55 (18%) patients. This is a higher proportion than in the Lonneux et al. study (8/233 patients) [32]. This is likely to be due to differences in the methodology by which a “change in radiotherapy planning” is defined. For example, in our series patients in whom an extra nodal level appears to have been included within the high-dose volume are classed as a change in radiotherapy planning.

The impact of FDG PET-CT upon T-stage assessment has not been assessed in this study. Tumour size assessment based upon FDG PET is highly dependant upon the method of segmentation and no superiority over conventional imaging has been demonstrated [9].

Refinements in imaging techniques are likely to lead to further improvements in the results obtained with functional imaging modalities. The results of FDG-PET-CT imaging are affected by differing acquisition protocols, image reconstruction, and interpretation techniques [40]. Standardisation of these processes will help with the comparison of the results of multicentre PET/CT series. A dedicated head and neck FDG PET-CT protocol may offer advantages in the detection of small lymph node metastases [41]; especially the use of intravenous contrast will allows the identification of small-volume and/or necrotic lymph nodes. Studies of novel PET tracers [16] may bring further improvements. Diffusion-weighted MRI has demonstrated encouraging results in the assessment of cervical lymph node metastases [42, 43].

## 5. Conclusion

In this series of patients with locally advanced HNSCC, FDG PET-CT altered the TNM stage and the identification of levels of nodal involvement in 31% and 40%, respectively. FDG PET-CT has potentially significant consequences for target volume definition in the IMRT era. The addition of imaging substudies to ongoing clinical trials will provide the ideal platform to optimise FDG PET-CT protocols and to further define a role in the management of HNSCC.

## Figures and Tables

**Table 1:** Disease site, subsite, stage, histology, and intended treatment prior to PET-CT examination.

Characteristic	Number (%)
Oropharynx Tonsil Base of tongue Posterior pharyngeal wall	47 (85%) 25 21 1
Hypopharynx Pyriform fossa	4 (7%) 4
Larynx Supraglottis	2 (4%) 2
Paranasal sinus	1 (2%)
Oral cavity Retromolar trigone	1 (2%) 1
Stage	
III IV	14 (25%) 41 (75%)
Histology	
SCC Poorly differentiated Moderately differentiated Well differentiated Not classified	55 (100%) 29 (53%) 18 (33%) 0 (0%) 8 (15%)
Intended treatment	
Radical radiotherapy	12 (22%)
Concurrent chemoradiotherapy	36 (65%)
Induction + concurrent chemoradiotherapy	6 (11%)
Concurrent cetuximab and radiotherapy	1 (2%)

**Table 2:** TNM distribution prior to PET-CT examination.

	T1	T2	T3	T4	
N0			4	2	6
N1	4	5	1	1	11
N2a	2	0	0	0	2
N2b	6	8	2	5	21
N2c	2	6	2	4	14
N3	1	0	0	0	1
	15	19	9	12	55

**Table 3:** Summary of TNM alterations following PET-CT.

Effect of PET-CT on TNM	No. of patients
Upstaging	11 (20%)
N0 to N1 N0 to N2b N0 to N2c N1 to N2c N2a to N2b N2b to N2c M0 to M1	1 1 2 1 1 4 1
Downstaging	6 (11%)
N1 to N0 N2c to N2a N2c to N2b	1 1 4
No impact	38 (69%)

**Table 4:** Summary of alterations in lymph node level involvement based on PET-CT (*n* = 22).

	Lymph node levels identified on clinical staging, MRI, and/or CT	Lymph node levels identified on PET-CT in combination with clinical staging, MRI, and/or CT	Alteration due to PET-CT
1	Right II	None	Downclassified equivocal LN
2	Left II, III, Right II	Left II, III	Downclassified equivocal contralateral level II LN
3	Left II, III	Left II, III, V	Identified sub-cm LN level V
4	Right II	Right II, III	Identified sub-cm LN level III
5	Left II, III, IV	Left II, III, IV, Right II	Identified contralateral 1 cm LN
6	Left II, Right II	Left II	Downclassified equivocal contralateral R II
7	None	RP	Identified RP involvement
8	Left II, IV	Left Ib, II, IV	Identified 1 cm LN level 1b
9	None	Right II, III. Left II, III	Identified bilat LN subcm
10	Left II, IV	Left II, III, IV	Identified 12 mm LN level III
11	Left II, III, IV, V, Right II	Left II, III, IV, V	Downclassified equivocal contralateral II
12	Right 1b-V	Right 1b-V, Left II	Identified equivocal contralateral level II
13	Right II, Left II	Right II	Downstages equivocal contralateral II
14	Right Ib, II, III Left Ib, II, III	Left II	Downstaged multiple equivocal LN
15	None	Right II, Left III	Identified sub-cm bilateral LN
16	None	Right III	Identified 2 sub-cm LN in level III
17	Right III	Right II, III	Identified 2 LN (1 cm) in level II
18	Right II, Left II, III	Right II, III, Left II, III, RP	Identified sub-cm LN level III and 13 mm RP LN
19	Right III	Right III, Left III	Identified 1 cm contralateral Level III
20	Left II, III, IV	Right II, left II, III, IV	Identified 1 cm contralateral level II
21	Right II, Left II	Right II, Left 1b, II	Identified 7 mm Ib
22	Right II, III, V	Right II, III, IV, V, Left RP	Identified sub-cm level IV and contralateral RP

**Table 5:** Impact of PET-CT upon radiotherapy planning in patients with altered identification of involved lymph node levels (*n* = 20).

	No of patients
PET-CT downclassified lymph node levels:	6
Lymph node not treated that is unilateral radiotherapy	1
Lymph node in prophylactic dose region	2
In high-dose region	2
Parallel opposed pair hence lymph node in high-dose region	1
Overall: likely plan change	3

PET-CT identified additional FDG-avid lymph node levels:	14
Lymph node in high-dose region	11
Lymph nodal level in prophylactic dose region	2
Parallel opposed pair hence lymph	1
node in high-dose region	
Overall: likely plan change	7
